# Societal economic burden of multiple sclerosis and cost-effectiveness of disease-modifying therapies

**DOI:** 10.3389/fneur.2022.1015256

**Published:** 2022-10-20

**Authors:** Steven Simoens

**Affiliations:** Department of Pharmaceutical and Pharmacological Sciences, KU Leuven, Leuven, Belgium

**Keywords:** multiple sclerosis, economic burden, costs, cost-effectiveness, disease-modifying therapies

## Abstract

**Background:**

In an era of scarce resources, policy makers, neurologists and other stakeholders need to be aware of the economic burden of multiple sclerosis and the cost-effectiveness of disease-modifying therapies. The aim of this article is to provide a mini-review of these health economic facets of multiple sclerosis.

**Methods:**

An umbrella review was conducted by searching PubMed and Google Scholar from 2002 until June 2022 for peer-reviewed systematic and narrative literature reviews.

**Results:**

An extensive body of evidence corroborates that multiple sclerosis is associated with a substantial economic burden within and outside the health care sector, that costs of secondary progressive multiple sclerosis exceed those of relapsing-remitting multiple sclerosis, that costs increase with disease severity and are influenced by the occurrence of relapses and therapy adherence. However, cost estimates and their breakdown into various components vary between countries. Economic evaluations show that disease-modifying therapies for relapsing-remitting multiple sclerosis are generally not cost-effective, but these results depend on the local setting. Cost-effectiveness of disease-modifying therapies improves when a societal perspective is taken and efficacy does not wane over a lifetime horizon, when oral administration forms or dosing strategies requiring less maintenance are introduced, and when generic versions enter the market. Reimbursement recommendations related to disease-modifying therapies also differ between countries.

**Conclusion:**

The local context matters when calculating the societal economic burden of multiple sclerosis and the cost-effectiveness of disease-modifying therapies.

## Introduction

A recent study investigated not only the effectiveness, but also the cost-effectiveness of 360 treatment sequences involving disease-modifying therapies (DMTs) in patients with relapsing-remitting multiple sclerosis (MS) in the Netherlands (1). The results indicated that the treatment sequence generating the highest health gain was not the same as the most cost-effective sequence. It is therefore important that policy makers, MS neurologists and other stakeholders are aware of the economic burden of MS and also consider evidence regarding the cost-effectiveness of DMTs in their decisions.

The aim of this article is to provide a mini-review of the health economics of MS by focusing on the costs that MS imposes on society, by exploring the cost-effectiveness of DMTs, and by examining the methodology of economic evaluations of DMTs. Although a review of literature reviews of MS cost-of-illness analyses was recently published (2), the added value of this article is the broader focus on multiple health economic facets of MS.

## Methods

In light of the many literature reviews examining the economic burden of MS and the cost-effectiveness of DMTs, this mini-review took the form of an umbrella review. This methodology is particularly suited to synthesize the state of the art of the evidence and to provide an overview of different facets of a research question (3).

PubMed and Google Scholar were searched until June 2022 using search terms related to MS (MS, clinically isolated syndrome, relapsing-remitting MS, primary progressive MS, secondary progressive MS), economic burden (cost-of-illness, health care costs, productivity loss, (in)direct (non-)medical costs), and economic evaluation (cost-effectiveness, cost-consequence, cost-utility, cost-benefit, value) alone and in combination with each other.

The search included all types of literature reviews, but excluded reviews that were published in abstract form only as these provide insufficient details. Literature reviews published since 2002 were considered given that previous evidence may not longer reflect current disease and its management. Reviews could be written in English, French, German or Dutch. No geographic search restrictions were applied.

## Results

The literature search generated ten reviews on societal costs associated with MS and 20 reviews on the cost-effectiveness of DMTs. These are discussed in the following sections.

### Societal economic burden of MS

**Figure 1:**
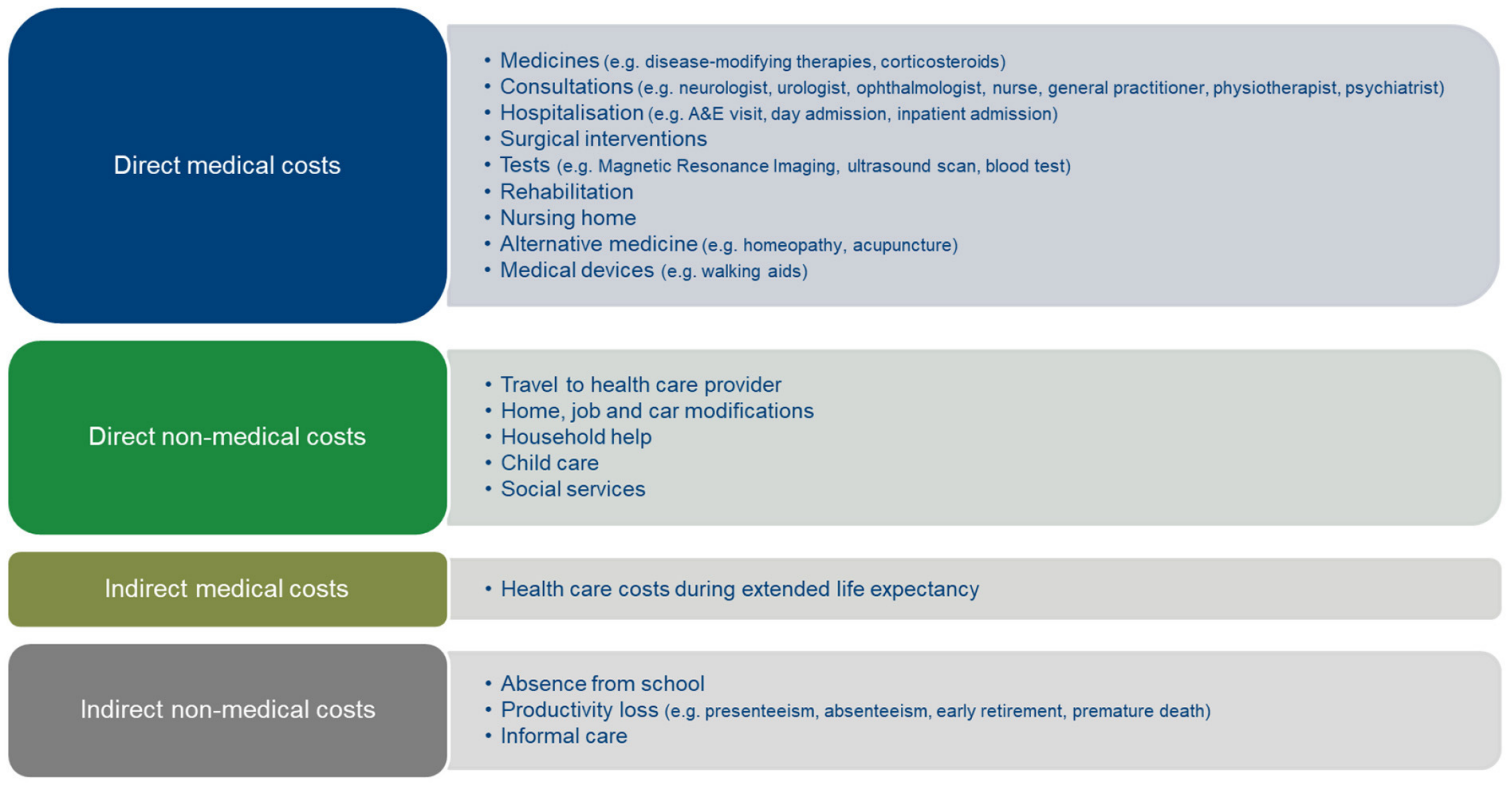
Cost components of the societal economic burden of MS. Author's figure based on Ernstsson et al. (4) and Fernandez et al. (5).

The societal economic burden of MS relates to how much and which costs that this disease generates within and outside the health care sector. Based on the literature (4, 5), Figure 1 lists the various cost components that need to be considered when calculating the societal economic burden of MS. This Figure distinguishes between direct medical costs, i.e., health care costs such as costs of disease-modifying therapies, neurologist consultations, rehabilitation and walking aids; non-medical costs directly associated with MS such as travel expenses and home modifications; indirect medical costs, i.e., health care costs associated with other diseases during extended life expectancy with MS treatment; and indirect non-medical costs or costs of productivity loss of MS patients and their informal caregivers.

Societal costs of MS are substantial and vary between countries. For instance, an analysis of 20 cross-sectional retrospective European cost-of-illness analyses found that mean annual societal costs per MS patient amounted to €40,303 (in 2015 values), but with notable inter-country variation (highest costs were observed in Western Europe and the lowest in Eastern Europe) (6). A breakdown of mean annual societal costs per MS patient based on 17 cost-of-illness analyses showed that the economic burden of MS is driven by costs of productivity loss (accounting for 39% of total costs), drug costs (21% of costs), and costs of informal caregivers (15% of costs) (7). However, a review of 23 mainly European cost-of-illness analyses also indicated that the relative importance of (in)direct (non-)medical cost components in societal MS costs differs between countries (8).

The literature suggests that geographic variation in size and breakdown of societal MS costs can be explained by contextual factors such as the organization and financing of a country's health care system, the availability and use of health care services, and regulation governing sickness and disability insurance and retirement (4, 6, 8–10). Additionally, cost estimates vary as a result of differences in study methodology between cost-of-illness analyses (4, 5, 9–11). Such differences may relate to the selection of the patient sample (e.g., MS type and severity level, evolution in MS diagnostic criteria over time), the use of a prevalence-based or incidence-based epidemiological approach, the application of a bottom-up or top-down cost measurement approach, data sources (e.g., patient questionnaires, medical records, claims database), the scope and categorization of cost components considered.

The review by Kolasa (7) also indicated that indirect costs associated with MS (accounting for 54% of societal costs) exceed direct costs. For instance, according to a meta-analysis of 23 cost-of-illness analyses conducted in Europe, North America or Asia, mean annual indirect costs per MS patient were US$20,167 (in 2014 values) (12). The identification of indirect costs as the main driver of the societal economic burden of MS originates from the early age of diagnosis and the lifelong duration of the disease (12). MS has a negative impact on employment, with workforce participation decreasing with higher disease severity. In this respect, a survey of 13,391 patients from 16 European countries noted that the employment rate decreased from 82% in MS patients with Expanded Disability Status Scale (EDSS) score 0 to 8% in patients with EDSS score 9 (9).

Disease severity plays a role in the societal economic burden of MS, influencing both the size and the breakdown of costs (4, 6, 8). As calculated by a review of 12 cost-of-illness analyses, mean annual societal costs amounted to US$22,719 (in 2011 values) per MS patient with mild disease severity (generally defined as EDSS score 0-3), US$40,153 per patient with moderate disease severity (generally EDSS score 4–6.5), and US$64,853 per patient with severe disease (generally EDSS score 7–9) (4). From a health economic perspective, it is therefore important to develop new therapies that influence disease severity or progression, as such therapies are more likely to be cost-effective. The same review also showed that the societal economic burden of MS mainly derives from drug costs in patients with mild disease severity and from productivity loss of patients and their informal caregivers when the disease is severe (4).

In addition to disease severity, the occurrence of relapses is an important driver of societal MS costs (8), as demonstrated for example by four Spanish cost-of-illness analyses (5). These studies not only indicated that costs per relapse are substantial, but cost estimates also vary by disease severity, country and MS center. Based on these findings, it is to be expected that therapies which reduce the number of MS relapses, have a higher probability of being cost-effective.

The societal economic burden varies between MS types: an analysis of seven European cost-of-illness analyses computed that mean annual costs per patient were €31,007 (in 2021 values) for relapsing-remitting MS and €58,475 for secondary progressive MS (13). With respect to their breakdown, indirect costs and direct non-medical costs accounted for more than half of societal costs of secondary progressive MS. Our literature search did not identify a review focusing on the economic burden of primary progressive MS.

Therapy adherence has also been shown to be a cost driver of MS (14): a narrative literature review concluded that adherence to disease-modifying therapies is associated with less resource use (i.e., hospitalisations, accident & emergency department visits) and lower health care costs (in addition to providing clinical benefits) (15).

Finally, whereas the previous literature reviews related to the economic burden of MS in high-income countries, fewer cost-of-illness analyses have been conducted in low- and middle-income countries. A recent analysis of 14 cost-of-illness analyses in upper-middle-income countries reported similar findings as in high-income countries: there is geographic variation in MS cost estimates, the economic burden of MS is greater when the disease is more severe, and the relative importance of cost components depends on disease severity (16).

### Cost-effectiveness of DMTs

The key question is whether DMTs for MS are cost-effective. A large number of well-conducted economic evaluations of DMTs exist in relapsing-remitting MS from the United States and Europe (10, 14, 17–24). Although literature reviews exploring the cost-effectiveness of DMTs were published over a 20-year period and clinical practice has evolved over time, the following conclusions were consistent across reviews. Results of economic evaluations varied, were sometimes conflicting, and the cost-effectiveness of specific therapies depended on the local setting. In general, the literature tended to conclude that DMTs are not cost-effective at commonly used willingness-to-pay thresholds. However, pegylated interferon and dimethyl fumarate were cost-effective in most economic evaluations, and increased efficacy and lower costs made ocrelizumab and alemtuzumab cost-effective. DMTs as compared with supportive care were more cost-effective than DMTs as compared to other active therapy. Early treatment of MS with DMTs dominated (i.e., was more effective and cheaper) than delayed treatment. Multiple literature reviews corroborated that administration route and frequency is an important DMT characteristic for MS patients, and showed that oral DMTs tend to be cost-effective as compared with injectable DMTs (22, 25). When focusing on the determinants of DMT cost-effectiveness, several reviews of economic evaluations indicated that cost-effectiveness results were most sensitive to changes in the effectiveness and acquisition prices of DMTs (22, 24, 26, 27).

The literature suggested that the cost-effectiveness of DMTs improves when the economic evaluation considers a lifetime horizon, when treatment efficacy does not wane over time, and when the analysis is conducted from a societal perspective (14, 17–21, 23, 24). Other factors that are likely to improve the cost-effectiveness of DMTs include: (a) lower DMT prices in Europe than in the United States; (b) discounts/rebates offered by pharmaceutical companies in the context of managed entry agreements; (c) the market entry of generic versions of for example glatiramer acetate; (d) the development of therapies with an oral administration form (e.g., cladribine); and (e) the introduction of dosing strategies requiring less maintenance (e.g., alemtuzumab) (14, 17–21). The literature also points to the off-label use of the effective and less expensive rituximab (biosimilar), but this has not been investigated in economic evaluations (28).

Caution needs to be exercised when interpreting these results on the cost-effectiveness of DMTs in light of methodological limitations of existing economic evaluations. Based on the literature (24, 26, 27, 29–31), Table 1 identifies several methodological challenges when calculating the cost-effectiveness of DMTs and provides recommendations on how to address these challenges. In particular, future economic evaluations need to draw on contemporary natural disease progression data, model the cost-effectiveness of DMT sequences, and account for the broader impact of MS interventions on patient well-being (24, 29, 31, 32).

**Table 1:** Methodological challenges and recommendations when calculating the cost-effectiveness of DMTs.

**Methodological issue**	**Challenge**	**Recommendation**
Technique of economic evaluation	Cost-minimization, cost-effectiveness, cost-utility or cost-benefit analysis	Apply cost-utility or cost-benefit analysis if difference in life expectancy and/or quality of life
Intervention and comparator	Majority of economic evaluations compare single DMT with supportive care	-Need to establish cost-effectiveness of DMT as compared with other active therapy;
		-Account for treatment discontinuation and consider treatment sequences;
		-Conduct multiple technology appraisal
Perspective	Significant cost impact of MS outside health care sector	Adopt societal perspective
Time horizon	Uncertainty about duration of treatment efficacy	Consider multiple time horizons in sensitivity analysis
Natural disease progression	-Evidence is dated and does not reflect actual clinical practice;	-Need for current, longitudinal studies of MS disease course;
	-Disease progression is measured by change in EDSS score and relapse occurrence	-Use country-specific MS registry;
		-EDSS does not capture cognitive, psychological and other patient-relevant outcomes
Mortality	General or MS-specific mortality	Use general population data adjusted for MS mortality risk
Utility values	Cost-effectiveness is likely to be sensitive to utility values	Use jurisdiction-specific utility values
Relative effectiveness of DMTs	Evidence mainly relates to efficacy of DMT vs. supportive care	-Need for RCTs comparing different DMTs;
		-Conduct network meta-analysis, simulated treatment comparison or matching-adjusted indirect comparison;
		-Collect RWE in actual clinical practice and use MS registries
Outcome measure	Intermediate measure (e.g., number of relapses avoided) or final measure (e.g., QALY)	Use QALYs
Modeling approach	Heterogeneous disease and treatment	Apply Markov model or carry out discrete event simulation
Model validity		Conduct and report activities exploring different types of model validity
Uncertainty	Cost-effectiveness is likely to be sensitive to changes in input parameter values	Conduct extensive deterministic and probabilistic sensitivity analyses

Author's table based on Hernandez et al. (24), Thompson et al. (26), Guo et al. (27), Wiyani et al. (29), Yamamoto and Campbell (30), and Hawton et al. (31).

DMT, disease-modifying therapy; EDSS, Expanded Disability Status Scale; MS, multiple sclerosis; QALY, quality-adjusted life year; RCT, randomized controlled trial; RWE, real-world evidence.

Many economic evaluations of DMTs are funded by pharmaceutical industry (29) in the context of a reimbursement application. A review of appraisals of DMTs for relapsing-remitting MS conducted by health technology assessment agencies in seven OECD countries found that reimbursement recommendations for the same product vary between agencies as a result of differences in how agencies assess cost-effectiveness and appraise evidence (33). Furthermore, this review showed that additional characteristics (e.g., unmet need, administration route and frequency) play a role in reimbursement recommendations. Finally, when comparing the cost-effectiveness of MS interventions (mainly DMTs) from a societal perspective vs. a health care payer perspective, a systematic literature review indicated that the consideration of productivity loss and informal care can change reimbursement recommendations (34).

## Discussion

In an era of scarce resources, attention needs to be paid to the societal costs associated with MS and to the cost-effectiveness of DMTs.

Although there is an extensive literature pointing to the substantial economic burden that MS imposes on society, there are several notable gaps in the current evidence base (4, 11, 12, 14). First, most cost-of-illness analyses calculate the burden over a specific time period (e.g., a year), but few studies employ an incidence-based approach which captures the lifelong and progressive nature of MS and relapses. As a step forward, a simulation exercise could estimate the lifetime economic burden of MS in a country based on mean annual societal costs per MS patient, the distribution of patients across MS types and severity levels, and the mean amount of time that a patient spends with a specific MS type and severity level. Second, although many cost-of-illness analyses consider productivity loss, this is usually limited to absenteeism and few analyses account for presenteeism or premature mortality. Third, there is a lack of evidence on the societal economic burden of primary progressive MS.

Cost-of-illness data can also be used in a creative way, for example, to identify patients at higher risk of developing MS. This is because a recent cost comparison between 1,988 MS patients and 7,981 matched persons without MS in Sweden indicated that MS patients have higher societal costs, health care costs and costs of productivity loss during the years prior to and following diagnosis, with the cost difference increasing over time (35). Although such an approach does not replace the use of diagnostic criteria, it may serve to trace MS patients at an earlier stage.

There is a voluminous literature investigating whether DMTs for MS are cost-effective (10, 14, 17–24). While this literature questions the cost-effectiveness of DMTs, it is important for policy and decision makers to note that cost-effectiveness results are specific to the local setting in which the economic evaluation is conducted. Moreover, the existing literature tends to under-estimate the cost-effectiveness of DMTs as it typically does not capture their impact on broader aspects of patient well-being (32). In this respect, the development of a new generic preference-based measure, the EQ-HWB (EQ Health and Wellbeing) (36), is timely and future research needs to explore its' usefulness in the context of MS. This also fits in a wider trend where regulatory authorities request data on patient-reported outcome measures and patient-reported experience measures when evaluating new drugs (37).

Economic evaluations of MS interventions tend to focus on DMTs, but less attention is paid to the cost-effectiveness of other interventions such as symptomatic therapies, psychotherapy or rehabilitation. With respect to these latter MS interventions, our literature search did not identify any review of economic evaluations.

## Conclusion

This mini-review of health economic facets of MS has demonstrated that policy makers, neurologists and other stakeholders need to base their decisions on local results when it comes to the economic burden of MS and the cost-effectiveness of DMTs. This is because, although studies consistently indicate that MS is associated with a substantial burden within and outside the health care sector, cost estimates and their breakdown into components vary between countries. Also, despite DMTs not being cost-effective in general, results depend on the local setting and the application of managed entry agreements, for example, is likely to improve the cost-effectiveness of these products.

## Author contributions

SS developed the idea and design of this study, carried out the review, and wrote the manuscript.

## Conflict of interest

This article is based on a presentation that Author SS gave at the physician educational webinar “Choosing the right DMD: impact on disease, QoL and societal cost”, hosted by Merck on 28th September 2021.

## Publisher's note

All claims expressed in this article are solely those of the authors and do not necessarily represent those of their affiliated organizations, or those of the publisher, the editors and the reviewers. Any product that may be evaluated in this article, or claim that may be made by its manufacturer, is not guaranteed or endorsed by the publisher.
